# A Complex Scenario of Nonsteroidal Anti-inflammatory Drugs Induced Prostaglandin E2 Production and Gut Microbiota Alteration in Clostridium difficile-Infected Mice

**DOI:** 10.1128/mBio.02596-19

**Published:** 2020-01-14

**Authors:** Maryam Noori, Abbas Yadegar, Mohammad Reza Zali

**Affiliations:** a Foodborne and Waterborne Diseases Research Center, Research Institute for Gastroenterology and Liver Diseases, Shahid Beheshti University of Medical Sciences, Tehran, Iran; b Gastroenterology and Liver Diseases Research Center, Research Institute for Gastroenterology and Liver Diseases, Shahid Beheshti University of Medical Sciences, Tehran, Iran; Vanderbilt University Medical Center

**Keywords:** NSAID, indomethacin, prostaglandin E2, *Clostridium difficile*, gut microbiota, 15-hydroxyprostaglandin dehydrogenase

## LETTER

We read with much interest the article by Maseda et al. entitled “Nonsteroidal Anti-inflammatory Drugs Alter the Microbiota and Exacerbate Clostridium difficile Colitis while Dysregulating the Inflammatory Response” (1). Today, C. difficile infection (CDI) is one the most prevalent nosocomial infections and a major concern for public health globally (2). Several risk factors, including antibiotic therapy, prolonged hospitalization, a weakened immune system, diet, advanced age, and an imbalanced gut microbiota, have emerged as modulators of CDI severity and risk (3, 4). Recent epidemiological data have demonstrated an association between the use of nonsteroidal anti-inflammatory drugs (NSAIDs) and CDI, but the mechanisms to explain this have not been elucidated (5).

As noted by the authors, treatment of mice with the NSAID indomethacin altered the proinflammatory profile and disrupted the intestinal barrier by perturbing epithelial cell junctions. They also showed that these effects were paralleled by specific alterations in the gut microbiota, which dramatically increased mortality and the intestinal pathology associated with CDI in mice. Interestingly, they observed that indomethacin pretreatment prevented downregulation of the *Ptgs1* and *Ptgs2* genes, encoding COX-1 and COX-2, respectively, and induced the expression of the *Ptges* gene and paradoxically increased the prostaglandin E2 (PGE2) concentrations upon CDI. The authors partly justified this paradox by stating that indomethacin reduced expression of the gene encoding the PGE2-inactivating enzyme called 15-hydroxyprostaglandin dehydrogenase (*Hpgd*, 15-PGDH) and suggested a “rebound” effect following temporary exposure to indomethacin prior to CDI. In contrast, several previous studies have strongly shown the stimulatory effects of NSAIDs, particularly indomethacin, on transcriptional and protein expression of 15-PGDH and its activity in different cell lines (6–9). In line with these findings, Munoz-Miralles et al. observed a significant reduction of PGE2 levels in cecal tissues of mice infected with C. difficile upon administration of indomethacin (10).

PGE2 is a metabolite of arachidonic acid and is synthesized by the cyclooxygenase (COX) enzymes. To date, three COX isoforms have been described: COX-1, COX-2, and COX-3. COX-1 is considered a housekeeping enzyme and is constitutively expressed in crypt epithelial cells, while COX-2 can be induced in a variety of cell types, including epithelial cells, macrophages, and fibroblasts. The COX-3 isoform is a splice variant of COX-1; however, there is much debate on the function of this enzyme. Notably, COX-2 can be rapidly induced upon exposure to various stimuli, such as proinflammatory cytokines, lipopolysaccharide (LPS), injury, and infectious agents (11). PGE2 mediates a variety of cellular processes and is able to exert pleiotropic effects in colorectal tumors, promoting proliferation, survival, angiogenesis, migration, and invasion mainly via the COX-2/PGE2 signaling pathway (12).

**FIG 1:**
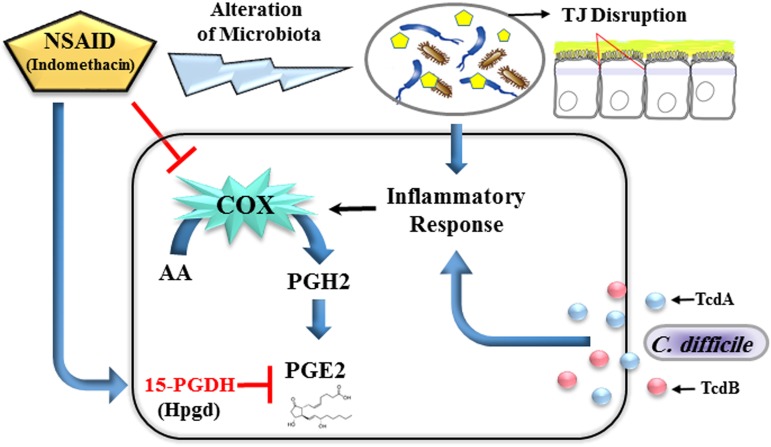
Possible scenario for impacts of the NSAID indomethacin on the up-modulation of 15-hydroxyprostaglandin dehydrogenase (*Hpgd*, 15-PGDH) expression and alteration of the gut microbiota, which disrupt the tight junctions (TJs) of intestinal epithelial cells to allow the translocation of bacteria and their metabolites into the bloodstream. In parallel, Clostridium difficile and its TcdA and TcdB toxins can induce the proinflammatory response that triggers the induction of inducible cyclooxygenase 2 (COX-2), leading to enhancement of prostaglandin E2 (PGE2) production.

Previous studies using animal models and humans suggest that C. difficile and its toxins induce the production of PGE2 (13–16). Kim et al. have shown that TcdA toxin induces COX-2 expression and releases PGE2 in a dose- and time-dependent manner (14). Moreover, Meyer et al. reported that TcdB also can directly stimulate human mast cells to synthesize PGE2/PGD2 in a p38 mitogen-activated protein kinase (MAPK)-dependent pathway (16). Taken together, we respectfully suggest, in disagreement with the conclusions of the Maseda et al. (1), that the NSAID indomethacin is able to induce 15-PGDH expression rather than its suppression, which may lead to elevated PGE2 production. Furthermore, we hypothesize that indomethacin-induced gut microbiota dysbiosis in association with a CDI-related proinflammatory profile can trigger the induction of inducible COX-2, which in turn enhances the production of PGE-2 (Fig. 1).
